# Multimorbidity and co-occurring musculoskeletal pain do not modify the effect of the selfBACK app on low back pain-related disability

**DOI:** 10.1186/s12916-022-02237-z

**Published:** 2022-02-08

**Authors:** Cecilie K. Øverås, Tom I. L. Nilsen, Barbara I. Nicholl, Guy Rughani, Karen Wood, Karen Søgaard, Frances S. Mair, Jan Hartvigsen

**Affiliations:** 1 Department of Public Health and Nursing, NTNU - Norwegian University of Science and Technology, Trondheim, Norway; 2 Department of Sports Science and Clinical Biomechanics, University of Southern Denmark, Odense, Denmark; 3 Institute of Health and Wellbeing, University of Glasgow, Glasgow, UK; 4 Department of Clinical Research, University of Southern Denmark, Odense, Denmark; 5 Chiropractic Knowledge Hub, University of Southern Denmark, Odense, Denmark

**Keywords:** Low back pain, Musculoskeletal pain, Comorbidity, Multimorbidity, Self-management, Telemedicine, Mobile applications, Digital technology, Artificial intelligence, Randomized controlled trial

## Abstract

**Background:**

selfBACK, an artificial intelligence (AI)-based app delivering evidence-based tailored self-management support to people with low back pain (LBP), has been shown to reduce LBP-related disability when added to usual care. LBP commonly co-occurs with multimorbidity (≥ 2 long-term conditions) or pain at other musculoskeletal sites, so this study explores if these factors modify the effect of the selfBACK app or influence outcome trajectories over time.

**Methods:**

Secondary analysis of a randomized controlled trial with 9-month follow-up. Primary outcome is as follows: LBP-related disability (Roland Morris Disability Questionnaire, RMDQ). Secondary outcomes are as follows: stress/depression/illness perception/self-efficacy/general health/quality of life/physical activity/global perceived effect. We used linear mixed models for continuous outcomes and logistic generalized estimating equation for binary outcomes. Analyses were stratified to assess effect modification, whereas control (*n* = 229) and intervention (*n* = 232) groups were pooled in analyses of outcome trajectories.

**Results:**

Baseline multimorbidity and co-occurring musculoskeletal pain sites did not modify the effect of the selfBACK app. The effect was somewhat stronger in people with multimorbidity than among those with LBP only (difference in RMDQ due to interaction, − 0.9[95 % CI − 2.5 to 0.6]). Participants with a greater number of long-term conditions and more co-occurring musculoskeletal pain had higher levels of baseline disability (RMDQ 11.3 for ≥ 2 long-term conditions vs 9.5 for LBP only; 11.3 for ≥ 4 musculoskeletal pain sites vs 10.2 for ≤ 1 additional musculoskeletal pain site); along with higher baseline scores for stress/depression/illness perception and poorer pain self-efficacy/general health ratings. In the pooled sample, LBP-related disability improved slightly less over time for people with ≥ 2 long-term conditions additional to LBP compared to no multimorbidity and for those with ≥4 co-occurring musculoskeletal pain sites compared to ≤ 1 additional musculoskeletal pain site (difference in mean change at 9 months = 1.5 and 2.2, respectively). All groups reported little improvement in secondary outcomes over time.

**Conclusions:**

Multimorbidity or co-occurring musculoskeletal pain does not modify the effect of the selfBACK app on LBP-related disability or other secondary outcomes. Although people with these health problems have worse scores both at baseline and 9 months, the AI-based selfBACK app appears to be helpful for those with multimorbidity or co-occurring musculoskeletal pain.

**Trial registration:**

NCT03798288. Date of registration: 9 January 2019

**Supplementary Information:**

The online version contains supplementary material available at 10.1186/s12916-022-02237-z.

## Background

Low back pain (LBP) is the leading cause of years lived with disability globally [1, 2] and is costly to societies due to sickness absence, lost productivity, and healthcare costs [3]. LBP is commonly accompanied by musculoskeletal (MSK) pain in other body regions [4], and people with LBP and co-occurring MSK pain are more likely to experience persistent and disabling pain with a poorer prognosis than those with LBP alone [5, 6]. LBP is also a ‘component disorder’ in multimorbidity [7]. Multimorbidity, the coexistence of two or more long-term conditions (LTCs) [8, 9], is a growing global challenge, with negative impacts on health care utilization [10], mortality [11], and quality of life [12]. Previous studies have found LBP to be associated with a range of other conditions such as anxiety and depressed mood, osteo- and rheumatoid arthritis, cardiovascular disease, diabetes, gastrointestinal, and respiratory disorders [13–25]. In primary care, multimorbidity prevalence varies from < 15% to > 95% depending on method of measurement, age, socioeconomic status, and gender [26]. Most primary care workload and hospital admissions involve people with multimorbidity [27], and when MSK pain is part of multimorbidity, it increases the impact on physical health and health care costs [28].

LBP guidelines recommend self-management strategies encouraging people to learn about and manage their condition, thereby supporting autonomy and independence [29, 30]. Advice on self-management may potentially be delivered by digital health interventions (websites, mobile applications, wearable technology) that are seen as scalable and feasible ways to engage care-seekers [31, 32]. However, despite rapid growth [33], the evidence for effectiveness, safety, and appropriateness of digital health interventions to support self-management in LBP remains weak [34].

A multinational randomized controlled trial (RCT) of the selfBACK digital intervention, designed to deliver evidence-based, individually tailored self-management support for people with LBP through an artificial intelligence (AI)-based app [35, 36], sought to improve the evidence base in this emerging area. The effect of the selfBACK system on reducing LBP-related disability showed overall small, but statistically significant benefits of the AI-based app when used in addition to usual care [37].

Assessing possible modifiers of effect was listed in the prespecified statistical analysis plan for the main trial. Multimorbidity and co-occurring MSK pain may contribute to a more complex clinical picture and resilience to treatment that could influence the effect of the selfBACK intervention. As part of the trial, data on the presence of LTCs and co-occurring MSK pain were collected, providing a unique opportunity to investigate whether people with multimorbidity and co-occurring MSK pain experience the same intervention effect when compared to those without. Additionally, using the trial population as a cohort allows for the study of trajectories of outcomes over time. Self-management is similarly recommended or even deemed necessary for people with multimorbidity [38], because multiple long-term conditions complicate care needs [39]. To our knowledge, no RCT on digital health interventions for LBP has studied the impact of multimorbidity on the clinical effectiveness of the intervention.

We aimed to address the following research questions: (1) Is the effect of the selfBACK system on LBP-related disability, quality of life, stress, depression, general health, illness perception, self-efficacy, physical activity, and global perceived effect, modified by baseline multimorbidity or number of co-occurring MSK pain sites? (2) Are baseline number of LTCs and co-occurring MSK pain sites associated with baseline measures and 9-month trajectories of these outcomes?

## Methods

The national ethical committees in Denmark (S-20182000-24) and Norway (2017/923-6) approved this trial, and all participants provided written informed consent. The trial is registered with ClinicalTrials.gov (NCT03798288).

### Study design, setting, participants and procedures

This paper presents secondary analyses of a single-blind randomized controlled trial among patients with non-specific LBP, randomized to two parallel groups to test the selfBACK system in addition to usual care (intervention arm) versus usual care only (control arm). The protocol for the selfBACK project and the selfBACK trial have been published [35, 36]. The selfBACK trial was conducted in Trondheim, Norway, and Odense, Denmark. Participants were recruited by physiotherapists, chiropractors, and general practitioners in primary care and from the Spine Centre outpatient spine clinic in Southern Denmark from March to December 2019. In brief, people aged 18 years or older seeking healthcare advice for non-specific LBP of any duration within the preceding 8 weeks that were interested in participating were screened for eligibility. Patients needed to score six or above on the Roland-Morris Disability Questionnaire (RMDQ), have access to a smartphone to install the selfBACK app, and have an email address to meet inclusion criteria. Exclusion criteria have been described in detail previously [36, 37].

After consenting to participation and completing a baseline questionnaire, block randomization of participants was performed in a web-based trial management system. In addition to usual care, the intervention group had access to the full content of the data-driven selfBACK system delivered via the AI-based selfBACK app and a connected physical activity-detecting wristband. Details of the selfBACK app content and procedures are described in greater detail elsewhere [35]. The app’s main elements are educational material, exercises for strength and flexibility, and tracking of physical activity (i.e. step count) detected by the wristband. Based on these three main elements, participants are presented with weekly individually tailored self-management plans to match the participant’s health status by case-based reasoning (CBR) technology, a branch of AI. The selfBACK app provided participants with instant feedback following individually set goals [35] likely to motivate and enhance participation in the trial. Participants in the control arm were asked to continue the care plan from their health care professional and whatever other help they found relevant.

For the first aim in this secondary analysis, the RCT design was used to explore effect modification in analyses stratified by multimorbidity status or number of co-occurring MSK pain sites. For the second aim, the control and intervention group were pooled and analysed according to number of LTCs and MSK pain sites.

### Data collection and variables

All participants completed the baseline web questionnaire at the start of the trial, and the pre-defined outcome variables were assessed at 6 weeks and 3, 6, and 9 months with 3 months as the primary follow-up time point.

Multimorbidity was defined as the coexistence of two or more LTCs (LBP + ≥ 1 LTC) [8, 9], versus no multimorbidity (LBP only). In the pooled sample, we further categorized people according to LTC count (LBP only, 1 LTC, ≥ 2 LTCs). The LTC variables were drawn from the baseline questionnaire applying questions adapted from the Norwegian HUNT study [40], covering the following categories: mental health issues, osteoarthritis, inflammatory arthritis, gastrointestinal problems, respiratory conditions, cardiovascular conditions, diabetes, neurological, cancer, and other LTCs.

Co-occurring MSK pain was defined as current pain marked on a mannequin with eight MSK pain site options in addition to LBP (neck, shoulders, upper back, elbows, lower back, wrists/hands, hips/thighs, knees, ankles/feet). This pain site mannequin is a modified version of the validated and commonly used standardized Nordic Questionnaire [41]. To assess possible effect modification, co-occurring MSK pain at baseline was classified according to number of sites additional to LBP (0–1, ≥ 2). To assess trajectories of outcomes from baseline and over the 9-month trial period in the pooled sample, we classified number of MSK pain sites according to the sample specific distribution of participants (LBP + 0–1 pain site, LBP + 2–3 pain sites, LBP + ≥ 4 pain sites).

The primary outcome was LBP-related disability measured using the RMDQ [42], where higher scores (0–24) indicate higher disability. Secondary outcomes were chosen based on availability of data guided by the recommendations in the Core Outcome Set for Multimorbidity Research (COSmm) [43]. This included health-related quality of life (EQ-5D: range 0–100, with higher scores indicating better health status) weighted according to Danish value set [44]; Perceived Stress Scale (PSS: range 0–40, where higher scores indicate greater perceived stress) [45]; Patient Health Questionnaire-8 (PHQ-8: 8-question 0–24 point scale, where higher score indicate greater depressive symptoms) [46]; General health (on a 100 point vertical scale, where higher score indicate better health) [47]; The Brief Illness Perception Questionnaire (BIPQ: range 0–10, where higher score indicate more threatening view of the pain) [48]; Pain Self-Efficacy Questionnaire (PSEQ: range 0–60, where higher scores reflect stronger confidence in ability to cope despite pain) [49]; Saltin-Grimby Physical Activity Level Scale assessing leisure time physical activity (4 levels of intensity collapsed into a binary variable sedentary/some physical activity and regular/hard physical activity) [50]; and Patient’s Global Perceived Effect (GPE: range − 5 to 5, where positive scores indicate LBP improvement and negative scores a worsening) [51].

### Statistical analysis

For research question 1, we estimated the effect of the intervention in strata according to multimorbidity status (no multimorbidity vs multimorbidity [LBP + ≥ 1 LTC]) or number of co-occurring MSK pains sites (≤ 1 additional pain site, ≥ 2 additional pain sites) using a constrained longitudinal data analysis approach, as described in the primary outcome paper [37]. In this approach, a linear mixed model was used for continuous outcomes and a logistic generalized estimating equation (GEE) model for binomial outcomes. Analyses were conducted according to the intention-to-treat principle, including all available data for all participants at each time point as specified in the statistical analysis plan of the main trial. Percentage of complete data at each time point can be found in the main trial paper [37]. Any missing values are inherently accounted for in the mixed model approach. Estimates of effect modification were calculated as the difference in the strata specific effects at 3 and 9 months.

To address research question 2, we presented trajectories of all outcomes from baseline to 6 weeks and 3, 6, and 9 months descriptively as means (SD). We further estimated crude mean changes from baseline to 9 months for all outcomes within categories of baseline multimorbidity status and number of co-occurring pain sites using linear mixed models including the adjusted difference in mean change from baseline between the categories with only LBP at baseline as the common reference. Logistic GEE models estimated odds ratio for physical activity at 3 and 9 months, comparing categories of multimorbidity and number of co-occurring pain sites. We justified pooling of the control and intervention group as the distribution of people with and without multimorbidity and categories of co-occurring MSK pain was evenly distributed due to random allocation among those who received the selfBACK app and not (multimorbidity status: no multimorbidity—only LBP 45.6% in control and 54.4% in intervention group; LBP + 1 LTC 56.9% in control and 43.1% in intervention group; LBP + ≥ 2 LTCs 47.0% in control and 52% in intervention group; number of co-occurring pain sites: LBP + ≤ 1 pain site 46.8% in control and 53.2% in intervention group; LBP + 2–3 pain sites 48.8% in control and 51.2% in intervention group; LBP + ≥ 4 pain sites 56.2% in control and 43.8% in intervention group).

Similar to the main paper [37], all effects were adjusted for variables used for the stratified randomization (i.e. country of recruitment [Denmark, Norway], clinical setting of recruitment [General Practitioner, Physiotherapist, Chiropractor, Outpatient back clinic]) and possible prognostic variables (age [years], sex [female, male], education [< 10 years, 10–12 years, > 12 years], duration of current pain episode at baseline [≤ 4 weeks, 5–12 weeks, > 12 weeks], and average pain intensity in the preceding week at baseline [0–10 scale]. Associations between categories of multimorbidity status and number of co-occurring pain sites (i.e. in the pooled sample) were additionally adjusted for workability index [0–10 scale], BMI [kg/m^2^], and physical activity level [self-reported four level Saltin-Grimby questionnaire]). The precision of all estimated associations is given by a 95% confidence interval. All analyses were performed using Stata version 16.1 (StataCorp LLC).

## Results

**Fig. 1:**
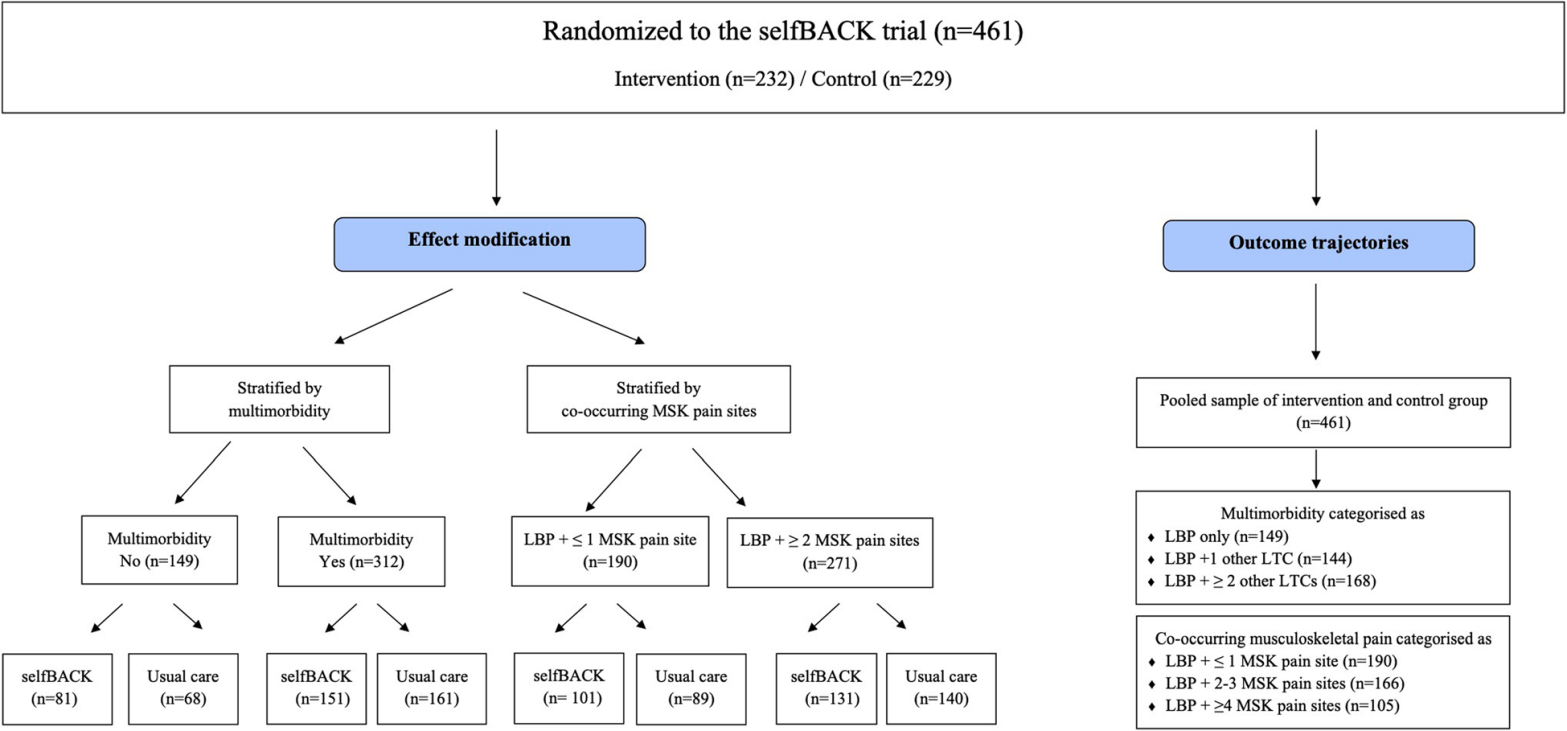
Flow of participants. LBP, low back pain; LTCs, long-term conditions; MSK, musculoskeletal

Among the 461 enrolled and randomly assigned participants, 229 were randomized to usual care (control arm) and 232 randomized to selfBACK adjunct to usual care (intervention arm). Of the LBP participants, 312 were categorized as having multimorbidity (LBP + ≥ 1LTC) and 271 had ≥ 2 co-occurring MSK pain sites in addition to LBP respectively. Full details are provided in Fig. 1.

People with multimorbidity were slightly older than those without, and people with multimorbidity or several co-occurring MSK pain sites were more often females, had somewhat higher pain intensity, and lower workability and physical activity levels. The majority had > 12 years of education (about 65%), and 60% had ≥ 12 weeks of LBP duration with no differences across the groups (Table 1).

**Table 1:** Baseline characteristics of the study population, stratified by study arm, multimorbidity status and number of co-occurring MSK pain sites

Variable	Multimorbidity				No. of co-occurring MSK pain sites			
	No (*n* = 149)		Yes (*n* = 312)		0-1 (*n* = 190)		2+ (*n* = 271)	
	Usual care (*n* = 68)	**self**BACK (*n* = 81)	Usual care (*n* = 161)	**self**BACK (*n* = 151)	Usual care (*n* = 89)	**self**BACK (*n* = 101)	Usual care (*n* = 140)	**self**BACK (*n* = 131)
Age, mean (SD), years	42.1 (13.9)	42.4 (13.0)	48.7 (14.2)	51.4 (15.1)	45.4 (15.6)	48.0 (14.7)	47.6 (13.6)	48.5 (15.3)
Women, %	50.0	44.4	62.1	56.3	49.4	47.5	64.3	55.7
Education: > 12 years, %	61.8	69.1	64.0	63.6	66.3	72.3	61.4	60.3
Multimorbidity, %	–	–	–	–	34.2	35.8	65.8	64.2
Co-occurring MSK pain (2+), %	24.3	25.9	75.7	74.1	–	–	–	–
Pain duration								
≤ 4 weeks, %	32.3	24.7	20.5	25.1	28.1	29.7	21.5	21.4
5–12 weeks, %	10.3	19.8	19.3	17.9	15.7	20.8	17.1	16.8
> 12 weeks, %	57.4	55.5	60.2	57.0	56.2	49.5	61.4	61.8
Pain intensity, mean (SD), NRS 0–10	4.5 (2.0)	4.6 (1.9)	5.1 (1.8)	5.0 (2.0)	4.9 (2.0)	4.6 (1.9)	5.0 (1.8)	5.0 (2.0)
Work ability index, mean (SD), 0–10	6.9 (1.8)	6.8 (2.2)	6.6 (1.8)	6.6 (1.8)	6.6 (1.9)	6.9 (2.0)	6.7 (1.8)	6.5 (1.9)
Body mass index, mean (SD), kg/m^2^	25.9 (4.3)	27.3 (4.6)	28.6 (5.6)	27.4 (4.8)	26.5 (4.6)	26.9 (4.6)	28.6 (5.7)	27.7 (4.9)
Physical activity								
Sedentary/some physical activity, %	51.5	46.9	64.6	63.0	53.9	55.5	65.0	58.8
Regular/hard physical activity, %	48.6	53.1	35.4	37.0	46.1	44.5	35.0	41.2

*Abbreviations*: *MSK* musculoskeletal, *SD* standard deviation, *NRS* numerical rating scale

**Fig. 2:**
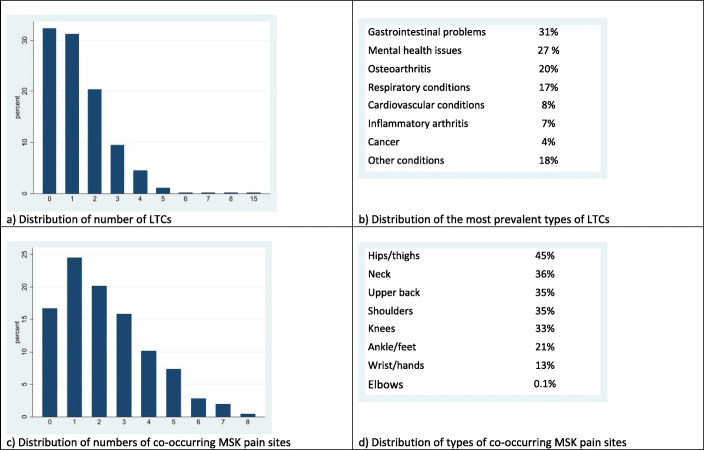
Distributions of number and types of LTCs and co-occurring MSK pain sites at baseline. LTCs, long-term conditions; MSK, musculoskeletal

The most frequent LTCs were gastrointestinal problems (31%) and mental health issues (27% [depression 18%; anxiety 9%]). At baseline, the mean number of LTCs were similar among those in the control and intervention arm (1.33 [SD 1.33] vs 1.32 [SD 1.54]). The most frequent co-occurring MSK pain sites alongside LBP were hips/thighs (45%). The mean number of co-occurring MSK pain sites was 2.34 (SD 1.8) in the control arm and 2.14 (SD 1.8) for the intervention arm (Fig. 2) (See Additional file 1: Table S1, for further details). There was no reported harm or adverse events by any participants.

### Effect modification of the intervention

**Fig. 3:**
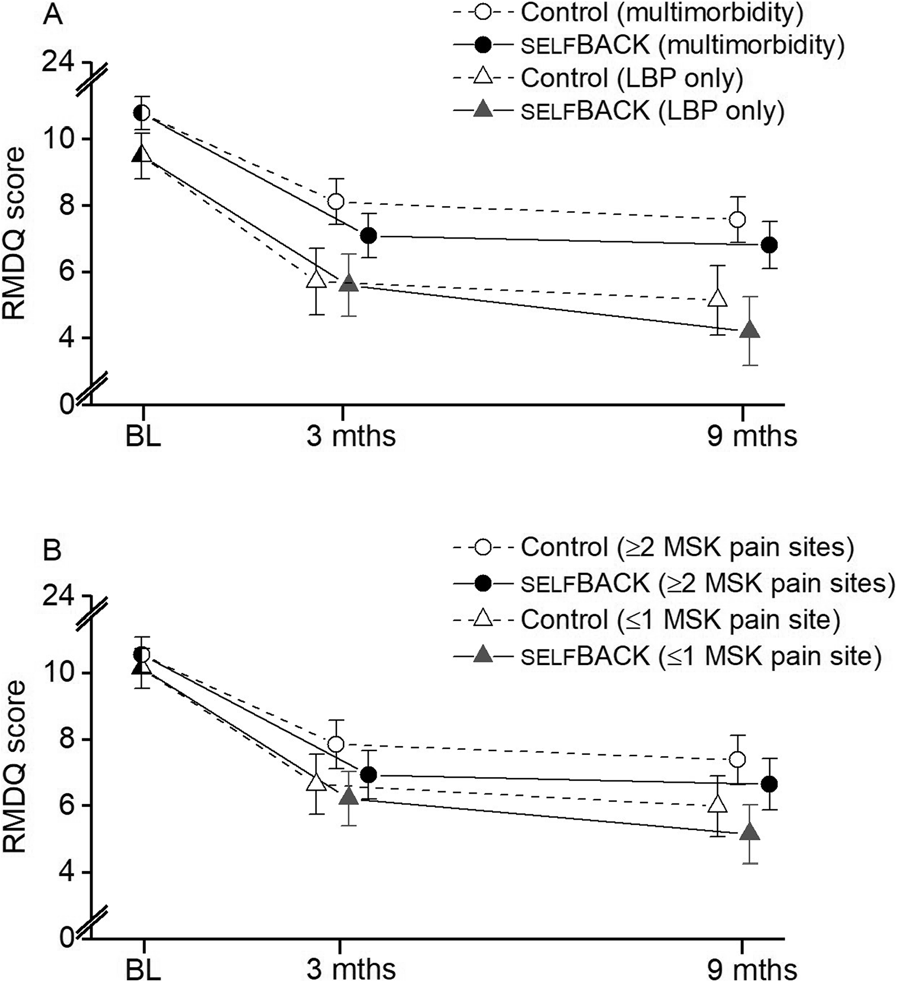
RMDQ scores and improvement at all time points for participants with and without multimorbidity and co-occurring MSK pain. RMDQ, Roland Morris Disability Questionnaire; MSK, musculoskeletal; LBP, low back pain

Overall, there was no clear evidence that multimorbidity modified the effect of the intervention on any of the outcomes under study. However, the adjusted mean difference in RMDQ score between the intervention and control arms at 3 months showed somewhat greater benefit for people with baseline multimorbidity, than for those without multimorbidity (mean difference due to interaction -0.9 [95 % CI − 2.5 to 0.6, *p* = 0.25]), while the effect resolved by 9 months follow-up (mean difference due to interaction 0.2 [95 % CI − 1.5 to 1.9, *p* = 0.81]). Having LBP and two or more additional MSK pain sites at baseline did not modify the effect at either three- or nine-month follow-up (Tables 2 and 3, Fig. 3).

**Table 2:** Effect of the selfBACK system on RMDQ and other core outcomes, stratified by multimorbidity status

	No multimorbidity (LBP only)			Multimorbidity (LBP + ≥1 LTC)			
	Mean (SD)^a^			Mean (SD)^a^			
Outcome	Usual care (*n* = 68)	**self**BACK (*n* = 81)	Adjusted^b^ mean difference (95% CI)	Usual care (*n* = 161)	**self**BACK (*n* = 151)	Adjusted^b^ mean difference (95% CI)	*P*_interaction_
RMDQ (0–24)							
Baseline	9.5 (4.4)			10.9 (4.4)			
3 months	5.7 (4.5)	5.7 (4.8)	− 0.1 (− 1.4 to 1.2)	8.2 (5.5)	7.2 (4.7)	− 1.0 (− 1.9 to − 0.2)	0.25
9 months	5.1 (4.5)	4.3 (4.9)	− 0.9 (− 2.3 to 0.5)	7.6 (5.8)	6.9 (5.3)	− 0.8 (− 1.7 to 0.1)	0.81
EQ-5D (0–1)							
Baseline	0.72 (0.14)			0.69 (0.12)			
3 months	0.78 (0.10)	0.77 (0.13)	− 0.00 (− 0.04 to 0.03)	0.73 (0.14)	0.75 (0.11)	0.02 (− 0.0 to 0.05)	0.25
9 months	0.82 (0.12)	0.83 (0.10)	0.01 (− 0.03 to 0.06)	0.73 (0.14)	0.76 (0.13)	0.03 (0.0 to 0.05)	0.66
PSS (0–40)							
Baseline	13.9 (6.9)			15.36 (6.8)			
3 months	13.8 (6.8)	12.6 (6.3)	− 1.1 (− 2.8 to 0.6)	15.2 (7.3)	14.5 (7.3)	− 0.6 (− 1.7 to 0.6)	0.68
9 months	11.6 (6.5)	11.0 (5.7)	− 0.5 (− 2.3 to 1.3)	14.7 (7.1)	13.0 (7.5)	− 1.6 (− 2.8 to − 0.4)	0.28
PHQ-8 (0–24)							
Baseline	5.7 (4.6)			6.8 (4.2)			
3 months	5.1 (4.3)	5.1 (4.3)	0.1 (− 1.0 to 1.1)	6.8 (4.6)	6.1 (4.6)	− 0.6 (− 1.3 to 0.1)	0.31
9 months	4.2 (4.4)	4.3 (3.6)	0.1 (− 1.0 to 1.3)	6.6 (4.9)	5.5 (4.4)	− 1.0 (− 1.8 to − 0.2)	0.10
General health (0–100)							
Baseline	69.3 (16.1)			64.7 (16.5)			
3 months	74.4 (15.6)	70.9(18.9)	− 3.0 (− 8.1 to 2.0)	69.0 (17.8)	70.9 (15.9)	1.8 (− 1.5 to 5.1)	0.10
9 months	78.5 (12.9)	77.7 (16.4)	− 0.6 (− 6.0 to 4.8)	69.0 (18.5)	71.4 (15.7)	2.2 (− 1.2 to 5.6)	0.39
BIPQ (0–80)							
Baseline	42.0 (11.0)			45.0 (10.7)			
3 months	37.9 (13.0)	34.7 (15.4)	− 3.3 (− 6.8 to 0.2)	41.5 (13.5)	36.3 (13.6)	− 5.0 (− 7.2 to − 2.9)	0.39
9 months	33.4 (17.2)	32.9 (12.6)	− 0.6 (− 4.4 to 3.1)	40.0 (13.3)	34.7 (15.8)	− 5.2 (− 7.4 to − 2.9)	0.04
PSEQ (0–60)							
Baseline	45.4 (10.7)			43.5 (11.2)			
3 months	48.9 (9.4)	50.7 (9.3)	1.8 (− 0.7 to 4.4)	45.6 (11.6)	48.4 (10.2)	2.7 (1.0 to 4.5)	0.57
9 months	48.9 (8.7)	52.7 (7.5)	3.8 (1.0 to 6.5)	46.0 (11.5)	49.1 (10.3)	2.9 (1.1 to 4.8)	0.59
GPE (− 5 to 5)							
3 months	1.5 (2.1)	1.9 (1.9)	0.4 (− 0.2 to 1.1)	1.2 (1.8)	2.0 (1.9)	0.8 (0.3 to 1.2)	0.35
9 months	2.0 (2.4)	2.4 (1.9)	0.5 (− 0.2 to 1.2)	1.1 (2.1)	2.1 (2.0)	0.9 (0.5 to 1.4)	0.27
	Odds ratio						
PA							
Baseline	–	–					
3 months	0.85	0.85	0.98 (0.53 to 1.84)	1.07	0.83	0.78 (0.50 to 1.21)	0.55
9 months	0.86	0.95	1.11 (0.56 to 2.22)	1.01	1.19	1.19 (0.72 to 1.94)	0.84

*Abbreviations*: *RMDQ* Roland Morris Disability Questionnaire, *LBP* low back pain, *LTCs* long-term conditions, *SD* standard deviation, *EQ-5D* health-related quality of life, *PSS* Perceived Stress Scale, *PHQ-8* Patient Health Questionnaire-8, *BIPQ* The Brief Illness Perception Questionnaire, *PSEQ* Pain Self-Efficacy Questionnaire, *GPE* Patient’s Global Perceived Effect, *PA* physical activity

^a^Marginal means from a crude linear mixed model, and SDs from raw data among persons with information at the specific time points

^b^Adjusted for country, recruiting clinician, education (< 10, 10–12, > 12 years), pain duration at baseline (≤ 4, 5–12, > 12 weeks), pain intensity as baseline (0–10 scale), sex (female vs male), and age (years)

**Table 3:** Effect of the selfBACK system on RMDQ and other core outcomes, stratified by number of co-occurring MSK pain sites

Outcome	0–1 co-occurring pain sites (LBP + ≤1 MSK pain site)			2+ co-occurring pain sites (LBP + ≥ 2 MSK pain sites)			
	Mean (SD)^a^			Mean (SD)^a^			
	Usual care (*n* = 89)	**self**BACK (*n* = 101)	Adjusted^b^ mean difference (95% CI)	Usual care (*n* = 140)	**self**BACK (*n* = 131)	Adjusted^b^ mean difference (95% CI)	*P*_interaction_
RMDQ (0–24)							
Baseline	10.2 (4.4)			10.6 (4.4)			
3 months	6.8 (5.0)	6.2 (4.5)	− 0.4 (− 1.6 to 0.7)	7.8 (5.6)	7.0 (4.9)	− 0.9 (− 1.9 to 0.0)	0.57
9 months	6.1 (5.1)	5.1 (4.5)	− 0.8 (− 2.0 to 0.4)	7.3 (5.8)	6.7 (5.7)	− 0.7 (− 1.7 to 0.3)	0.82
EQ-5D (0–1)							
Baseline	0.71 (0.15)			0.70 (0.11)			
3 months	0.76 (0.13)	0.78 (0.12)	0.01 (− 0.03 to 0.04)	0.73 (0.13)	0.74 (0.11)	0.02 (− 0.01 to 0.04)	0.71
9 months	0.78 (0.15)	0.80 (0.12)	0.02 (− 0.02 to 0.05)	0.74 (0.13)	0.77 (0.13)	0.03 (− 0.00 to 0.05)	0.71
PSS (0–40)							
Baseline	13.8 (6.5)			15.6 (7.0)			
3 months	13.4 (6.7)	12.3 (6.4)	− 0.7 (− 2.2 to 0.8)	15.7 (7.3)	15.0 (7.3)	− 0.7 (− 1.9 to 0.5)	0.98
9 months	12.4 (6.6)	10.9 (6.6)	− 1.2 (− 2.8 to 0.4)	14.7 (7.3)	13.4 (7.3)	− 1.3 (− 2.6 to − 0.0)	0.88
PHQ-8 (0–24)							
Baseline	5.5 (4.0)			7.0 (4.5)			
3 months	5.0 (3.9)	4.7 (3.7)	− 0.2 (− 1.1 to 0.7)	7.0 (4.9)	6.5 (4.9)	− 0.5 (− 1.3 to 0.3)	0.62
9 months	4.7 (4.9)	4.1 (3.7)	− 0.3 (− 1.3 to 0.6)	6.6 (4.8)	5.8 (4.6)	− 0.9 (− 1.7 to − 0.0)	0.43
General health (0–100)							
Baseline	68.7 (15.4)			64.4 (17.0)			
3 months	71.3 (18.4)	71.6 (17.3)	− 0.0 (− 4.2 to 4.2)	70.0 (16.9)	70.5 (16.6)	0.8 (− 2.9 to 4.4)	0.73
9 months	72.9 (18.3)	77.1 (14.7)	3.6 (− 0.8 to 8.1)	71.1 (17.7)	70.7 (17.0)	0.0 (− 3.8 to 3.9)	0.27
BIPQ (0–80)							
Baseline	43.5 (11.3)			44.4 (10.5)			
3 months	39.8 (13.9)	35.9 (14.1)	− 3.7 (− 6.6 to − 0.8)	40.8 (13.3)	35.7 (14.3)	− 5.3 (− 7.7 to − 2.8)	0.41
9 months	37.9 (16.3)	32.8 (13.7)	− 4.8 (− 7.8 to − 1.8)	38.1 (14.0)	35.1 (15.8)	− 3.2 (− 5.7 to − 0.7)	0.45
PSEQ (0–60)							
Baseline	45.2 (10.9)			43.3 (11.1)			
3 months	47.2 (11.4)	50.0 (9.1)	2.4 (0.2 to 4.7)	46.1 (11.0)	48.6 (10.5)	2.5 (0.6 to 4.5)	0.99
9 months	47.8 (10.9)	51.9 (8.3)	3.7 (1.3 to 6.1)	46.3 (11.1)	49.0 (10.4)	2.8 (0.8 to 4.9)	0.57
GPE (− 5 to 5)							
3 months	1.4 (2.1)	2.0 (2.0)	0.7 (0.1 to 1.3)	1.2 (1.8)	1.9 (1.8)	0.7 (0.2 to 1.2)	0.85
9 months	1.5 (2.2)	2.5 (1.9)	1.1 (0.5 to 1.7)	1.3 (2.2)	1.9 (2.1)	0.6 (0.1 to 1.1)	0.26
	Odds ratio						
PA							
Baseline	–	–		–	–		
3 months	1.03	0.83	0.82 (0.47 to 1.43)	0.96	0.85	0.89 (0.55 to 1.44)	0.80
9 months	0.78	0.82	1.09 (0.61 to 1.97)	1.09	1.37	1.29 (0.75 to 2.21)	0.65

*Abbreviations*: *RMDQ* Roland Morris Disability Questionnaire, *MSK* musculoskeletal, *LBP* low back pain, *SD* standard deviation, *EQ-5D* health-related quality of life, *PSS* Perceived Stress Scale, *PHQ-8* Patient Health Questionnaire-8, *BIPQ* The Brief Illness Perception Questionnaire, *PSEQ* Pain Self-Efficacy Questionnaire, *GPE* Patient’s Global Perceived Effect, *PA* physical activity

^a^Marginal means from a crude linear mixed model, and SDs from raw data among persons with information at the specific time points

^b^Adjusted for country, recruiting clinician, education (< 10, 10–12, > 12 years), pain duration at baseline (≤ 4, 5–12, > 12 weeks), pain intensity as baseline (0–10 scale), sex (female vs male), and age (years)

### Baseline differences and trajectories of outcomes stratified by LTC count and number of MSK pain sites

Baseline LBP-related disability (RMDQ) was higher for those with multimorbidity (LBP + ≥ 2 LTCs 11.3; LBP + 1 LTC 10.4; LBP only 9.5), and with more co-occurring MSK pain (LBP + ≥4 pain sites 11.3; LBP + ≥2–3 pain sites 10.1; LBP + ≤ 1 pain site 10.2). The secondary outcomes at baseline similarly showed higher baseline scores for stress, depression, higher illness perception, poorer pain self-efficacy, and lower general health ratings with more LTCs and co-occurring MSK pain. Quality of life measured by EQ5D was similar across the groups at baseline (Additional file 1: Table S2 and S3).

RMDQ gradually improved over time for all groups. However, participants with ≥ 2 LTCs experienced less reduction when compared to those with no or only one LTC in addition to LBP (adjusted mean difference at 9 months 1.5 [95% CI 0.5 to 2.5]). Similarly, for those with co-occurring MSK pain sites we saw that those with LBP and ≥ 4 pain sites improved less compared to those with fewer co-occurring pain sites (difference between groups 2.2 [95% CI 1.1 to 3.3] at 9 months).

For the secondary outcomes, all groups reported minimal improvement for all outcomes at all time points. Those with no LTCs and LBP with only 0–1 additional MSK pain site improved most on measures of stress, depression, and general health, while those with LBP and two or more LTCs and four or more additional MSK pain sites had less improvement of illness perception, self-efficacy, and the global perceived effect (see Additional file 1: Table S2 and S3, for details). For the health-related quality of life (EQ5D), the minimal improvements over time were similar across all groups. Those without LTCs and least co-occurring MSK pain were more physically active at baseline based on self-reported measures in the Saltin-Grimby questionnaire; with no change over the project period (Additional file 1: Table S4).

## Discussion

This secondary analysis showed that across different outcomes including LBP-related disability, the effect of the selfBACK intervention was similar regardless of baseline multimorbidity or co-occurring MSK pain status. In the pooled sample, those with multimorbidity and co-occurring MSK pain at baseline had slightly higher RMDQ score, and higher scores for stress and depression, as well as lower scores for illness perception, self-efficacy, and general health, when compared to those with no multimorbidity and fewer sites of co-occurring MSK pain. Participants with multimorbidity and co-occurring MSK pain all gradually improved in LBP-related disability over time with no difference between subgroups.

The fact that multimorbidity and co-occurring MSK pain did not modify the effect of the selfBACK intervention may indicate that the selfBACK system and potentially digital health interventions more broadly could be effective as a supplement to usual care regardless of health condition status. This aligns with a recent meta-analysis that proposed a positive role for mHealth-based self-management programs for persistent LBP [52]. Still, we expected weaker effect of the intervention among people with multimorbidity and co-occurring MSK pain. There could be several factors that explains why this was not observed in our data. It is plausible that the selfBACK system may enhance the capacity of people with multimorbidity to self-manage, lessening perceived treatment burden [53]. Closer personalized monitoring may be the essence that overrides the additional burden of engaging in multiple treatments and digital health barriers felt by some with multimorbidity [54]. Several behavioural change techniques like goal setting, getting feedback on the outcome, self-monitoring, coping planning, and development of autonomy that are highlighted in the literature [55–57] are integrated in the selfBACK app, and these may be critical elements that help people with LBP, co-occurring MSK pain and multimorbidity to self-manage. In addition, the fact that the advice is regarded as trustworthy and validated by health-care professionals could facilitate the use of LBP digital health interventions [58].

Our findings that adults with multimorbidity and co-occurring MSK pain experience more adverse effects at baseline than those without also aligns with previous literature which has shown that such individuals generally report higher levels of disability [4, 6, 59, 60], mental health problems [61–63], poorer workability [6, 7, 64, 65], physical inactivity, and obesity [5, 66, 67]. With this complex clinical picture and the cumulative burden of all these conditions, it is not surprising that people with multimorbidity also report higher illness perception, poorer pain self-efficacy, and lower general health. Lower socioeconomic status is associated with poorer health outcomes [68] and a lower level of education is associated with higher likelihood of multimorbidity [69]. However, in this study, about 65% of participants reported more than 12 years of education, perhaps reflecting the characteristics of people that health-care professionals chose to refer as potential participants to a trial. Hence generalizing to people with other health and social profiles should be undertaken with caution.

### Strengths and limitations

A major strength of this analysis is the inclusion of a range of LTCs, nine MSK pain sites, and outcome measures matching the international experts’ consensus on important core outcomes for multimorbidity intervention studies [43]. People with multimorbidity are often excluded from trials [70], so a key strength of this study was the inclusion of such a large proportion of people with multimorbidity, which broadens the applicability of the findings to the wider population seen in everyday clinical practice. Additionally, interventions for multimorbidity integrated within the healthcare system are suggested to be more effective but seldom evaluated [71]. Importantly, participants ranged in age from 18 to 86 years which is unusual for digital health interventions which typically exclude older adults [34].

Despite the relatively large sample included in the main RCT, the study was not powered for robust secondary analyses. The results may therefore be prone to random error and should be viewed as hypothesis-generating findings that need further examination in future studies. Moreover, all participants with multimorbidity and other MSK pain issues were eligible for inclusion, but it is still possible that health-care professionals may have selected individuals they considered more suitable for the trial, resulting in the inclusion of those who have greater digital health literacy or healthier individuals with multimorbidity and MSK pain. Another limitation was that diseases were self-reported and not clinically confirmed diagnoses, and we do not know anything about the severity of the conditions and whether some of them may be more significant in relation to LBP. We further counted LBP as one LTC when considering effect modification stratified by multimorbidity status as LBP commonly features in multimorbidity clusters [72], but this could be a weakness as about 25% reported a pain duration of ≤ 4 weeks. Our justification was that these participants most likely have persistent fluctuating LBP [73] which still fits within a LTC definition. Additionally, care-seekers tend to have recurrent LBP with a higher impact score [74], and hence possibly those experiencing their first episode of acute LBP were less likely to be referred into the trial.

### Implications and future research

This study shows that while people with multimorbidity or co-occurring musculoskeletal pain had greater pain related disability at baseline, this did not markedly modify the effects of the selfBACK app. Although the results of this subgroup analyses have limited statistical power, they do not indicate any large differences or harmful effects of the intervention. This has important implications for clinical practice as it suggests that people with LBP and additional long-term conditions or co-occurring MSK pain at other parts of the body could be encouraged to engage with such individually tailored digitally supported self-management interventions as it is possible that they will improve wellbeing.

Future research on AI-based mHealth relating to LBP and self-management could include multimorbidity and other MSK pain sites in the AI algorithms for tailoring advice, support, and exercises. It could further target modifiable risk factors that these conditions have in common [75]. An app that supports self-management of the range of LTCs that someone lives with may be a person-centred and time-efficient approach. Furthermore, as multimorbidity is not limited to older adults [76, 77], future trials should, as in this trial, investigate if mHealth can support care across age spans due to essential issues around workability, quality of life, and healthcare costs savings [75]. Importantly, frailty often co-exists with multimorbidity, especially in more socioeconomically deprived populations [78, 79], and the risk of disability is greater in such individuals. Therefore, future research should consider the applicability of digital self-management interventions for participants affected by frailty (with or without co-existing multimorbidity).

This study followed up people over 9 months, which is longer than most digital health studies targeting those with LBP; however, the longer sustainability of such interventions merits investigation [34]. The process evaluation of the selfBACK app will provide us with further information about user experiences, including barriers and facilitators to normalization of such interventions into everyday life [80]. Such additional insights will enhance our understanding of the implications of mHealth in the long-term management of people with LBP and multimorbidity. We need better insights to what different people consider a worthwhile effect for an easily accessible digital health intervention with limited risk such as the selfBACK app, until then it is uncertain whether the results are clinically meaningful for all.

## Conclusions

The effect of the selfBACK app was similar across a range of outcomes regardless of baseline multimorbidity or co-occurring MSK pain, suggesting that personalized AI-based apps for self-management of LBP can be considered in addition to usual care for those with multimorbidity or co-occurring MSK pain. The more complex the clinical picture and the larger the cumulative burden of additional LTCs or MSK pain, the more affected the participants were at baseline. However, multimorbidity did not affect the course of LBP as all subgroups gradually improved levels of LBP-related disability over time.

## Supplementary Information

**Additional file 1: Tables S1-S4**. **Table S1** – Baseline multimorbidity and co-occurring pain in study population. **Table S2** – Baseline multimorbidity and change for outcomes for all participants in the selfBACK trial. **Table S3** – Baseline numbers of co-occurring MSK pain sites and change for outcomes for all participants in the selfBACK trial. **Table S4** – Odds ratio for an increase in physical activity as assessed by the four-level Saltin-Grimby questionnaire.

## Data Availability

The data that support the findings of this study are available from Professor Paul Jarle Mork (paul.mork@ntnu.no) at the Norwegian University of Science and Technology (NTNU).

## Abbreviations

LBP: Low back pain
MSK: Musculoskeletal
LTCs: Long-term conditions
RCT: Randomized controlled trial
AI: Artificial intelligence
RMDQ: Roland-Morris Disability Questionnaire
CBR: Case-based reasoning technology
EQ-5D: Health-related quality of life
PSS: Perceived stress scale
PHQ-8: Patient Health Questionnaire-8 (depression)
BIPQ: The Brief Illness Perception Questionnaire
PSEQ: Pain Self-Efficacy Questionnaire
GEE: Generalized estimating equation
