# Supplementary material for: Multimorbidity and co-occurring musculoskeletal pain do not modify the effect of the selfBACK app on low back pain-related disability
Source: BMC Med. 2022 Feb 8;20:53. doi: 10.1186/s12916-022-02237-z (PMC8822859; doi:10.1186/s12916-022-02237-z)
Supplement: Supplementary file 1 — Additional file 1: Tables S1-S4. Table S1 – Baseline multimorbidity and co-occurring pain in study population. Table S2 – Baseline multimorbidity and change for outcomes for all participants in the selfBACK trial. Table S3 – Baseline numbers of co-occurring MSK pain sites and change for outcomes for all participants in the selfBACK trial. Table S4 – Odds ratio for an increase in physical activity as assessed by the four-level Saltin-Grimby questionnaire. [file 12916_2022_2237_MOESM1_ESM.docx]

**Supplementary tables**

**Supplementary Table S1** Baseline multimorbidity and co-occurring pain in study population

| **Long-term comorbidities to LBP** | **Overall N (%)** |
| --- | --- |
| **Long term conditions** |  |
| Gastrointestinal problems | 142 (30.8) |
| Mental health issues (Depression 85 [18.5%]/anxiety 40[8.7%]) | 125 (27.1) |
| Osteoarthritis | 93 (20.2) |
| Respiratory conditions (Asthma 64 [13.9%]/chronic bronchitis/emphysema/COPD 16 [3.5%]) | 80 (17.4) |
| Cardiovascular conditions  (Cardiovascular disease 27 [5.9%]/heart failure 2 [0.4%]/stroke/brain haemorrhage 6[1.3%]) | 35 (7.6) |
| Inflammatory arthritis  (Rheumatoid arthritis 12[2.6%]/psoriatic arthritis/psoriasis 21[4.6%], gout 1 [0.2]) | 34 (7.4) |
| Cancer | 20 (4.3) |
| Diabetes | 16 (3.5) |
| Neurological (epilepsy 5 [1.1%], trigeminal neuralgia 1 [0.2%], Tourettes syndrome 1 [0.2%],  Parkinson 1 [0.2%], Migraine 3 [0.7%], cluster headache 1 [0.2%]) | 12 (2.6) |
| Other LTCs (including sleep problems [sleep apnea, restless legs syndrome or narcolepsy] 25  [5.4%], kidney disease 11 [2.4], osteoporosis 7 [1.5%]) | 53 (11.5) |
| **Number of LTCs** |  |
| 0 | 149 (32.4) |
| 1 | 144 (31.3) |
| 2 | 93 (20.3) |
| 3 | 44 (9.6) |
| 4 | 21 (4.6) |
| 5 | 5 (1.1) |
| 6 | 1 (0.2) |
| 7 | 1 (0.2) |
| 8 | 1 (0.2) |
| 15 | 1 (0.2) |
| **Co-occurring MSK pain sites** |  |
| Hips/thighs | 206 (44.7) |
| Neck | 168 (36.4) |
| Upper back | 162 (35.1) |
| Shoulders | 161 (34.9) |
| Knees | 152 (33.0) |
| Ankel/feet | 97 (21.0) |
| Wrist/Hands | 59 (12.8) |
| Elbows | 28 (6.1) |
| **Number of co-occurring pain sites** |  |
| 0 (only LBP) | 77 (16.7) |
| 1 | 113 (24.5) |
| 2 | 93 (20.2) |
| 3 | 73 (15.8) |
| 4 | 47 (10.2) |
| 5 | 34 (7.4) |
| 6 | 13 (2.8) |
| 7 | 9 (2.0) |
| 8 | 2 (0.4) |

Abbreviations: LTCs, long term conditions; MSK, musculoskeletal; LBP, low back pain

^a^ Categorization based on 17 disease condition alternatives plus free text

**Supplementary Table S2** Baseline multimorbidity and change for outcomes for all participants in the selfBACK trial

|  | Mean (SD)^a^ | | | | |  | Change from baseline to 9 months | |
| --- | --- | --- | --- | --- | --- | --- | --- | --- |
| Outcome variables | Baseline | 6  wks | 3  mths | 6  mths | 9  mths |  | Crude mean  (95% CI) | Adj. difference ^b^  (95% CI) |
| RMDQ (0-24) |  |  |  |  |  |  |  |  |
| Only LBP | 9.5  (4.4) | 6.6 (4.7) | 5.7  (4.7) | 5.0  (4.6) | 4.7  (4.7) |  | -4.8 (-5.6 to -4.1) | 0.0 (Reference) |
| LBP + 1 LTC | 10.4 (4.1) | 7.0  (4.6) | 6.7  (4.8) | 6.2  (5.1) | 6.4  (5.0) |  | -4.0 (-4.7 to -3.3) | 0.8 (-0.2 to 1.9) |
| LBP + ≥2 LTCs | 11.3 (4.5) | 8.7  (5.3) | 8.4  (5.2) | 8.1  (5.6) | 8.0  (5.9) |  | -3.3 (-4.0 to -2.6) | 1.5 (0.5 to 2.5) |
|  |  |  |  |  |  |  |  |  |
| EQ5D (0-1) |  |  |  |  |  |  |  |  |
| Only LBP | 0.72  (0.14) | 0.76  (0.11) | 0.78  (0.12) | 0.80  (0.11) | 0.82  (0.11) |  | 0.10 (0.08 to 0.12) | 0.0 (Reference) |
| LBP + 1 LTC | 0.70  (0.12) | 0.76  (0.10) | 0.76  (0.13) | 0.77  (0.12) | 0.76  (0.13) |  | 0.06 (0.04 to 0.08) | -0.04 (-0.07 to -0.01) |
| LBP + ≥2 LTCs | 0.69  (0.12) | 0.72  (0.11) | 0.72  (0.12) | 0.74  (0.13) | 0.72  (0.13) |  | 0.03 (0.01 to 0.05) | -0.07 (-0.10 to -0.04) |
|  |  |  |  |  |  |  |  |  |
| PSS (0-40) |  |  |  |  |  |  |  |  |
| Only LBP | 13.9  (6.9) | 13.3  (5.9) | 13.1  (6.6) | 13.4  (7.1) | 11.3  (6.1) |  | -2.6 (-3.6 to -1.6) | 0.0 (Reference) |
| LBP + 1 LTC | 14.5  (6.2) | 13.9  (6.9) | 13.7  (7.0) | 12.7  (7.5) | 12.6  (7.1) |  | -1.9 (-2.8 to -0.9) | 0.7 (-0.7 to 2.0) |
| LBP + ≥2 LTCs | 16.1  (7.1) | 15.3  (7.2) | 15.8  (7.5) | 15.6  (7.2) | 15.0  7.2) |  | -1.1 (-2.0 to -0.2) | 1.4 (0.1 to 2.8) |
|  |  |  |  |  |  |  |  |  |
| PHQ-8 (0-24) |  |  |  |  |  |  |  |  |
| Only LBP | 5.7  (4.6) | 4.8  (3.9) | 5.1  (4.3) | 5.0  (4.4) | 4.2  (4.0) |  | -1.4 (-2.1 to -0.8) | 0.0 (Reference) |
| LBP + 1 LTC | 6.1  (3.7) | 5.3  (3.7) | 5.6  (4.1) | 5.3  (4.4) | 5.0  (4.0) |  | -1.1 (-1.7 to -0.5) | 0.4 (-0.5 to 1.2) |
| LBP + ≥2 LTCs | 7.4  (4.5) | 7.3  (5.2) | 7.1  (5.0) | 6.8  (4.8) | 6.9  (5.1) |  | -0.5 (-1.0 to 0.1) | 1.0 (0.1 to 1.8) |
|  |  |  |  |  |  |  |  |  |
| General health (0-100) |  |  |  |  |  |  |  |  |
| Only LBP | 69.2  (16.1) | 71.1  (17.5) | 72.5  (17.6) | 73.3  (17.5) | 78.0  (14.7) |  | 8.8 (5.8 to 11.7) | 0.0 (Reference) |
| LBP + 1 LTC | 65.7  (17.2) | 69.1  (17.7) | 71.4  (16.7) | 73.8  (15.6) | 71.4  (19.0) |  | 5.6 (2.7 to 8.5) | -3.0 (-7.1 to 1.1) |
| LBP + ≥2 LTCs | 63.8  (15.9) | 66.3  (18.3) | 68.7  (17.0) | 69.6  (16.5) | 69.2  (15.5) |  | 5.4 (2.7 to 8.1) | -3.3 (-7.3 to 0.6) |
|  |  |  |  |  |  |  |  |  |
| BIPQ (0-80) |  |  |  |  |  |  |  |  |
| Only LBP | 42.0  (11.0) | 36.6  (13.0) | 36.2  (14.4) | 35.1  (13.6) | 33.1  (15.1) |  | -8.9 (-10.9 to -7.0) | 0.0 (Reference) |
| LBP + 1 LTC | 45.0  (9.8) | 38.9  (11.5) | 37.6  (12.5) | 36.7  (13.9) | 36.5  (13.8) |  | -8.5 (-10.3 to -6.6) | 0.5 (-2.2 to 3.2) |
| LBP + ≥2 LTCs | 45.0  (11.4) | 41.2  (13.4) | 39.9  (15.2) | 38.9  (14.4) | 38.2  (15.8) |  | -6.8 (-8.6 to -5.1) | 2.1 (-0.5 to 4.7) |
|  |  |  |  |  |  |  |  |  |
| PSEQ (0-60) |  |  |  |  |  |  |  |  |
| Only LBP | 45.4  (10.7) | 49.0  (9.1) | 49.9  (9.3) | 49.5  (9.6) | 50.8  (8.3) |  | 5.4 (3.9 to 6.9) | 0.0 (Reference) |
| LBP + 1 LTC | 45.0  (9.9) | 47.3  (10.8) | 48.7  (9.8) | 49.5  (10.5) | 49.  (9.5) |  | 4.8 (3.3 to 6.2) | -0.7 (-2.8 to 1.4) |
| LBP + ≥2 LTCs | 42.2  (12.0) | 44.5  (11.9) | 45.6  (11.8) | 46.1  (11.6) | 45.9  (12.0) |  | 3.4 (2.0 to 4.8) | -2.0 (-4.0 to 0.0) |
|  |  |  |  |  |  |  |  |  |
| GPE (-5 to 5) |  |  |  |  |  |  |  |  |
| Only LBP | - | 1.6  (1.8) | 1.7  (2.0) | 1.9  (2.2) | 2.2  (2.2) |  | 2.2 (1.8 to 2.5) | 0.0 (Reference) |
| LBP + 1 LTC | - | 1.4  (1.8) | 1.5  (2.2) | 1.6  (2.3) | 1.8  (2.0) |  | 1.8 (1.4 to 2.1) | -0.4 (-0.9 to 0.2) |
| LBP + ≥2 LTCs | - | 1.2  (1.8) | 1.7  (1.7) | 1.4  (2.2) | 1.4  (2.2) |  | 1.4 (1.1 to 1.8) | -0.6 (-1.1 to -0.1) |

Abbreviations: SD, standard deviation; RMDQ, Roland Morris Disability Questionnaire; LBP, low back pain; LTCs, long term conditions; EQ-5D, Health-related quality of life; PSS, Perceived Stress Scale; PHQ-8, Patient Health Questionnaire-8; BIPQ, The Brief Illness Perception Questionnaire; PSEQ, Pain Self-Efficacy Questionnaire; GPE, Patient’s Global Perceived Effect.

^a^ Marginal means from a crude linear mixed model, and SDs from raw data among persons with information at the specific time points.

^b^ Adjusted for country, recruiting clinician, education (<10, 10-12, >12 years), pain duration at baseline (≤4, 5-12, >12 weeks), pain intensity as baseline (0-10 scale), sex (female vs male), age (years), body mass index (kg/m^2^), physical activity level at baseline (self-reported four level Saltin-Grimby questionnaire), and workability index at baseline (0-10 scale).

**Supplementary Table S3** Baseline numbers of co-occurring MSK pain sites and change for outcomes for all participants in the selfBACK trial

|  | Mean (SD)^a^ | | | | |  | Change from baseline to 9 months | |
| --- | --- | --- | --- | --- | --- | --- | --- | --- |
| Outcome variables | Baseline | 6  wks | 3  mths | 6  mths | 9  mths |  | Crude mean  (95% CI) | Adj. difference^b^  (95% CI) |
| RMDQ (0-24) |  |  |  |  |  |  |  |  |
| LBP + ≤1 pain site | 10.2  (4.4) | 7.0  (4.9) | 6.5  (4.8) | 6.3  (5.1) | 5.6  (4.9) |  | -4.6 (-5.3 to -4.0) | 0.0 (Reference) |
| LBP + 2-3 pain sites | 10.1  (4.1) | 7.1  (4.6) | 6.4  (4.9) | 5.7  (5.1) | 5.9  (5.5) |  | -4.2 (-4.9 to -3.5) | 0.4 (-0.5 to 1.4) |
| LBP + ≥4 pain sites | 11.3  (4.7) | 9.2  (5.3) | 9.1  (5.5) | 8.8  (5.4) | 8.9  (5.9) |  | -2.4 (-3.3 to -1.5) | 2.2 (1.1 to 3.3) |
|  |  |  |  |  |  |  |  |  |
| EQ5D (0-1) |  |  |  |  |  |  |  |  |
| LBP + ≤1 pain site | 0.71  (0.15) | 0.76  (0.10) | 0.77  (0.13) | 0.79  (0.12) | 0.79  (0.13) |  | 0.08 (0.06 to 0.10) | 0.0 (Reference) |
| LBP + 2-3 pain sites | 0.72  (0.09) | 0.75  (0.11) | 0.76  (0.12) | 0.78  (0.11) | 0.78  (0.12) |  | 0.07 (0.05 to 0.09) | -0.01 (-0.04 to 0.01) |
| LBP + ≥4 pain sites | 0.68  (0.12) | 0.70  (0.12) | 0.70  (0.13) | 0.71  (0.13) | 0.70  (0.13) |  | 0.02 (-0.00 to 0.05) | -0.06 (-0.09 to -0.03) |
|  |  |  |  |  |  |  |  |  |
| PSS (0-40) |  |  |  |  |  |  |  |  |
| LBP + ≤1 pain site | 13.8  (6.5) | 12.8  (6.3) | 12.8  (6.6) | 12.0  (6.7) | 11.6  (6.6) |  | -2.2 (-3.0 to -1.3) | 0.0 (Reference) |
| LBP + 2-3 pain sites | 14.6  (6.9) | 14.5  (6.7) | 14.5  (7.4) | 14.0  (7.6) | 12.9  (7.4) |  | -1.7 (-2.6 to -0.8) | 0.5 (-0.7 to 1.7) |
| LBP + ≥4 pain sites | 17.3  (6.8) | 16.3  (7.1) | 16.6  (7.1) | 17.2  (7.0) | 15.9  (7.0) |  | -1.3 (-2.5 to -0.2) | 0.8 (-0.7 to 2.2) |
|  |  |  |  |  |  |  |  |  |
| PHQ-8 (0-24) |  |  |  |  |  |  |  |  |
| LBP + ≤1 pain site | 5.5  (4.0) | 4.9  (4.2) | 4.8  (3.8) | 4.6  (3.9) | 4.4  (4.3) |  | -1.2 (-1.7 to -0.6) | 0.0 (Reference) |
| LBP + 2-3 pain sites | 6.2  (4.0) | 5.8  (4.4) | 6.2  (4.9) | 5.7  (4.7) | 5.6  (4.6) |  | -0.7 (-1.3 to -0.1) | 0.5 (-0.3 to 1.3) |
| LBP + ≥4 pain sites | 8.3  (5.0) | 7.9  (4.7) | 7.8  (4.7) | 7.8  (4.7) | 7.3  (4.7) |  | -1.0 (-1.7 to -0.3) | 0.2 (-0.7 to 1.1) |
|  |  |  |  |  |  |  |  |  |
| General health (0-100) |  |  |  |  |  |  |  |  |
| LBP + ≤1 pain site | 68.7  (15.4) | 71.3  (17.3) | 71.5  (17.8) | 74.2  (16.) | 75.0  (16.6) |  | 6.4 (3.8 to 8.9) | 0.0 (Reference) |
| LBP + 2-3 pain sites | 66.5  (16.4) | 69.4  (18.0) | 72.4  (16.1) | 74.5  (14.7) | 74.8  (15.4) |  | 8.3 (5.6 to 11.0) | 2.1 (-1.5 to 5.8) |
| LBP + ≥4 pain sites | 61.1  (17.5) | 62.7  (18.2) | 66.6  (17.4) | 64.7  (18.5) | 64.3  (18.7) |  | 3.2 (-0.3 to 6.7) | -3.0 (-7.4 to 1.3) |
|  |  |  |  |  |  |  |  |  |
| BIPQ (0-80) |  |  |  |  |  |  |  |  |
| LBP + ≤1 pain site | 43.5  (11.3) | 37.6  (12.9) | 37.6  (14.4) | 36.7  (14.2) | 35.2  (15.5) |  | -8.3 (-9.9 to -6.6) | 0.0 (Reference) |
| LBP + 2-3 pain sites | 43.5  (11.0) | 38.7  (13.2) | 36.5  (14.0) | 35.4  (13.7) | 34.7  (15.3) |  | -8.7 (-10.5 to -7.0) | -0.5 (-3.0 to 1.9) |
| LBP + ≥4 pain sites | 45.9  (9.7) | 42.2  (11.4) | 41.2  (13.8) | 40.3  (14.1) | 39.9  (14.0) |  | -6.0 (-8.3 to -3.8) | 2.2 (-0.6 to 5.1) |
|  |  |  |  |  |  |  |  |  |
| PSEQ (0-60) |  |  |  |  |  |  |  |  |
| LBP + ≤1 pain site | 45.2  (10.9) | 48.2  (10.7) | 48.8  (10.4) | 49.9  (9.9) | 49.9  (9.9) |  | 4.7 (3.4 to 6.0) | 0.0 (Reference) |
| LBP + 2-3 pain sites | 45.0  (10.5) | 48.3  (10.4) | 49.6  (9.8) | 49.0  (11.3) | 49.7  (10.2) |  | 4.7 (3.3 to 6.0) | -0.0 (-1.9 to 1.9) |
| LBP + ≥4 pain sites | 40.6  (11.4) | 41.9  (11.0) | 43.7  (11.5) | 44.3  (10.9) | 44.2  (11.4) |  | 3.6 (1.8 to 5.4) | -1.2 (-3.4 to 1.0) |
|  |  |  |  |  |  |  |  |  |
| GPE (-5 to 5) |  |  |  |  |  |  |  |  |
| LBP + ≤1 pain site | - | 1.5  (1.7) | 1.7  (2.1) | 1.9  (2.2) | 2.0  (2.1) |  | 2.0 (1.7 to 2.4) | 0.0 (Reference) |
| LBP + 2-3 pain sites | - | 1.5  (1.9) | 1.7  (1.8) | 1.6  (2.2) | 1.7  (2.3) |  | 1.7 (1.3 to 2.0) | -0.2 (-0.7 to 0.2) |
| LBP + ≥4 pain sites | - | 0.8  (1.6) | 1.3  (1.9) | 1.1  (2.2) | 1.4  (2.0) |  | 1.4 (0.9 to 1.8) | -0.4 (-1.0 to 0.1) |

Abbreviations: MSK, musculoskeletal; SD, standard deviation; RMDQ, Roland Morris Disability Questionnaire; LBP, low back pain; EQ-5D, Health-related quality of life; PSS, Perceived Stress Scale; PHQ-8, Patient Health Questionnaire-8; BIPQ, The Brief Illness Perception Questionnaire; PSEQ, Pain Self-Efficacy Questionnaire; GPE, Patient’s Global Perceived Effect.

^a^ Marginal means from a crude linear mixed model, and SDs from raw data among persons with information at the specific time points.

^b^Adjusted for country, recruiting clinician, education (<10, 10-12, >12 years), pain duration at baseline (≤4, 5-12, >12 weeks), pain intensity as baseline (0-10 scale), sex (female vs male), age (years), body mass index (kg/m^2^), physical activity level at baseline (self-reported four level Saltin-Grimby questionnaire), and workability index at baseline (0-10 scale).

**Supplementary Table S4** Odds ratio for an increase in physical activity as assessed by the four-level Saltin-Grimby questionnaire

|  | Proportion physically active | | | | | Adjusted odds ratio (95% CI)^a^ | |
| --- | --- | --- | --- | --- | --- | --- | --- |
|  | BL | 6 wks | 3 months | 6 months | 9 months | 3 months | 9 months |
| LTCs | | | | | | | |
| No | 51% | 53% | 47% | 48% | 48% | 1.0 (reference) | 1.0 (reference) |
| Yes, LBP + 1 LTC | 41% | 38% | 39% | 39% | 46% | 0.8 (0.5 to 1.4) | 1.1 (0.6 to 1.8) |
| Yes, LBP + ≥ 2 LTC | 32% | 35% | 31% | 26% | 30% | 0.7 (0.4 to 1.2) | 0.7 (0.4 to 1.2) |
|  |  |  |  |  |  |  |  |
| No. of co-occurring MSK pain sites | | | | | | | |
| 0-1 | 45% | 47% | 43% | 42% | 38% | 1.0 (reference) | 1.0 (reference) |
| 2-3 | 40% | 42% | 36% | 36% | 48% | 0.8 (0.5 to 1.3) | 1.5 (0.9 to 2.5) |
| 4+ | 34% | 30% | 35% | 29% | 33% | 0.9 (0.5 to 1.7) | 1.1 (0.6 to 2.0) |

Abbreviations: LTCs, long term conditions; LBP, low back pain; MSK, musculoskeletal.

Physical activity level dichotomised: 0=Level 1/2: Sedentary/ Some physical activity; 1=Level 3/4: Regular/ regular hard physical activity

^a^Adjusted for country, recruiting clinician, education (<10, 10-12, >12 years), pain duration at baseline (≤4, 5-12, >12 weeks), pain intensity as baseline (0-10 scale), sex (female vs male), age (years), body mass index (kg/m^2^), physical activity level at baseline (self-reported four level Saltin-Grimby questionnaire), and workability index at baseline (0-10 scale).
